# Six-year follow-up of the treatment of patients with dissociative disorders study*

**DOI:** 10.1080/20008198.2017.1344080

**Published:** 2017-06-28

**Authors:** Amie C. Myrick, Aliya R. Webermann, Richard J. Loewenstein, Ruth Lanius, Frank W. Putnam, Bethany L. Brand

**Affiliations:** ^a^ Family & Children’s Services, Bel Air, MD, USA; ^b^ University of Maryland, Baltimore County, Catonsville, MD, USA; ^c^ Sheppard Pratt Health Systems, Towson, MD, USA; ^d^ University of Western Ontario, London, ON, Canada; ^e^ University of North Carolina, Chapel Hill, NC, USA; ^f^ Towson University, Towson, MD, USA

**Keywords:** Dissociative disorders, long-term treatment, treatment, revictimization

## Abstract

**Objective**: Literature on the treatment of dissociative disorders (DDs) suggests that these individuals require long-term and specialized treatment to achieve stabilization and functionality. There is considerable empirical support for specialized phasic, dissociation-focused treatment in reducing a myriad of psychological symptoms and self-harm in this population. However, until recently, there has been a paucity of longitudinal treatment research on DD patients.

**Method**: In the present six-year follow-up study, 61 therapists who participated in the initial phase of the Treatment of Patients with Dissociative Disorders (TOP DD) study answered questionnaires about their study patient’s stressors, quality of life, global functioning, victimization, and safety. These results provided a view of patients’ progress six years since the beginning of the TOP DD study.

**Results**: Longitudinal analyses demonstrated patients had significantly fewer stressors (*Χ^2^*(6) = 18.76, *p *< .01, canonical *r *= .48, *N* = 76), instances of sexual revictimization (*X* ^2^(1) = 107.05, *p* < .001) and psychiatric hospitalizations (*t*(54) = 2.57, *p* < .05, Cohen’s *d* = .43), as well as higher global functioning (*Χ^2^*(2) = 59.27, *p *< .001, canonical *r *= .65, *N* = 111).

**Conclusions**: These findings continue to support the initial results of the TOP DD study that, despite marked initial difficulties and functional impairment, DD patients benefit from specialized treatment.

Patients with dissociative disorders (DDs) suffer from severe psychiatric symptoms, complex emotional, social, and physical health difficulties, and high levels of functional impairment related to chronic, severe childhood trauma (Foote, Smolin, Kaplan, Legatt, & Lipschitz, 2006; Johnson, Cohen, Kasen, & Brook, 2006; Putnam, 1997; Sar, AkyüZ, & Doğan, 2007). The severity and chronicity of patients’ impairment underscores the importance of studying the burden of disease and effective DD treatments. Reviews and meta-analyses have found that trauma-focused treatment can be particularly beneficial for adult survivors of childhood trauma, including DD patients (Brand, Classen, McNary, & Zaveri, 2009; Sachsse, Vogel, & Leichsenring, 2006). Inpatient treatment studies have found that specialized treatment programmes reduce posttraumatic, dissociative, interpersonal, and general psychiatric problems among dissociative patients (Ali & Smartt, 2009; Jepsen, Bad, Langeland, Sexton, & Heir, 2014; Jepsen, Svagaard, Thelle, McCullough, & Martinsen, 2009; Lampe, Hofmann, Gast, Reddemann, & Schüßler, 2014; Rosenkranz & Muller, 2011; Steil, Dyer, Priebe, Kleindienst, & Bohus, 2011). Outpatient DD treatment is also associated with enduring gains over time, including decreased dissociative, depressive and posttraumatic symptoms, self-destructiveness, and symptoms of comorbid disorders, as well as increased adaptive functioning (see Brand et al., 2009). Longitudinal studies have been conducted with DD patients, with durations of 1–10 years (Coons & Bowman, 2001; Coons & Millstein, 1986; Ellason & Ross, 1997; Jepsen et al., 2014, 2009; Kluft, 1984/1985; Lampe et al., 2014). These studies found that patients who remain in treatment generally show improvement in social, psychological, and occupational functioning.

The prospective, longitudinal Treatment of Patients with Dissociative Disorders (TOP DD) study is the largest treatment outcome study conducted with DD patients. TOP DD followed a large, international naturalistic sample of DD patients treated by outpatient community therapists. Results were consistent with previous studies in demonstrating the beneficial outcomes of DD treatment. (For a summary of study findings, see Supplemental data Table 1). Over 30 months, therapists reported decreases in patients’ dissociative, depressive, and posttraumatic symptoms; decreased incidence of self-harm; suicide attempts, drug use, and hospitalization; and increases in productive and social activities (Brand et al., 2013). In the current study, TOP DD therapists report on patients’ safety, quality of life, global functioning, interpersonal victimization, and stressors six years after having enrolled in the study.

**Table 1.** Summary of therapist participants.

	Therapists still treating TOP DD Patients	Therapists with TOP DD Patients who Terminated from Treatment	Therapists with Patients in Early Stage of Treatment (Stages 1–2)	Therapists with Patients in Late Stage of Treatment (Stages 3–5)	Total Sample^a^
*N*	63	39	17	44	102
%	61.7%	38.2%	16.67%	43.13%	

^a^ Two therapists’ data excluded from analyses because stage of treatment was not included. Sample included in analyses (*N* = 61)

## 1. Methods

### 1.1. Participants

Participants at the six-year follow-up (T5) included 61 clinicians enrolled in the original TOP DD study; details on recruitment, eligibility, and methodology are available (Brand et al., 2013). Any therapist who provided baseline (T1) data was contacted by email in September 2013, approximately six years from the time recruitment for the TOP DD study began, and was invited to complete a brief, web-based survey. TOP DD patients were not contacted to participate due to minimal contact information and concern that some patients no longer in treatment might experience distress about reporting on their current status. This study received IRB approval from Towson University and Sheppard Pratt Health System.

The TOP DD study initially collected data at four time points (T1–T4) over 30 months. Sample sizes for clinicians at each time point were as follows: T1 *N* = 295; T2 *N *= 189 (64% retention); T3 *N* = 174 (59% retention); T4 *N* = 135 (46% retention). At T1, clinicians in the current study reported that they had worked with their patients for an average of 6.5 years (*SD *= 4.5, range = 2–21 years).

### 1.2. Procedure

After providing informed consent, therapists indicated whether their TOP DD patients were still in treatment with them. A total of 102 therapists (35.4% of therapist T1 sample) responded to the T5 survey. Sixty-eight (66.67%) had completed surveys at all four previous data points. Of these, 63 had TOP DD patients still in treatment. Therapists who were no longer seeing their patients reported on the reason(s) patients were no longer in treatment and provided study feedback. They were not invited to answer subsequent questions. Therapists who were continuing to see their patients answered questions about patients’ current level of functioning and were required to provide their patients’ treatment stage to be included in study analyses. Of these patients, 4.9% were in stage 1 (*N* = 3), 23% were in stage 2 (*N* = 14), 18% were in stage 3 (*N* = 11), 24.6% were in stage 4 (*N* = 15), and 29.5% were in stage 5 (*N* = 18). Two therapists did not list their patients’ treatment stage, yielding a final T5 sample of *N* = 61 (21% retention) (see Table 1).

### 1.3. Measures

#### 1.3.1. Patient treatment stage

Clinicians reported patients’ therapeutic stage, following expert guidelines on stage-oriented treatment of complex trauma and dissociation (International Society for the Study of Trauma and Dissociation [ISSTD], 2011). Stages included 1 (i.e. stabilization and establishing safety), 3 (i.e. processing trauma memories) and 5 (i.e. integration and reconnection within self and with others). Patient stage was dichotomized into early stage (stages 1–2) and late stage (stages 3–5) and was controlled in analyses as a potential confounding variable.

#### 1.3.2. Patient stressors

At T2 through T5, clinicians reported the degree to which stressors negatively impacted the patient’s functioning and treatment over the previous six months using a Likert scale (0 = non-applicable, 3 = somewhat, 5 = highly negative impact). Stressors were *revictimization-related* (sexual revictimization, physical and emotionally abusive relationships), *family-related* (family of origin, marriage, and children), *therapy-oriented* (mistrust of therapist and treatment team), *resistance-related* (therapeutic resistance among self-states^1^ and fear of change), and *resource-related* (work, money, health, and housing). In order to increase power, stressors were merged into six composite stressor scores, for each five types of stressors as well as a total stressor score.

#### 1.3.3. Quality of life

At T1 through T5, clinicians reported the quality of patients’ romantic relationships, friendships, and employment status using a Likert scale (1 = lower/poor/unstable to 5 = higher/stable). In order to increase power, these variables were merged into a composite quality of life (QOL) variable.

#### 1.3.4. Global assessment of functioning

At T1 through T5, clinicians rated patients’ global functioning (GAF; American Psychiatric Association, 2000) using a Likert scale (1 = 1–10 on GAF, 2 = 11–20, 3 = 21–30, etc.), with higher scores indicating greater psychosocial functioning, safety, and stability of symptoms.

#### 1.3.5. Safety

At T1–T5, clinicians reported on patients’ number of admissions to inpatient hospitals, as well as number of days hospitalized, within the previous six months. Clinicians reported patients’ number of suicide attempts and self-injurious behaviours using a scale from 1–4 (1 = none, 2 = one, 3 = two to three, 4 = four or more) and 0–4 (0 = none, 2 = occasionally, 3 = frequently, 4 = daily or almost daily), respectively. Due to the low frequencies, these variables were dichotomized to three variables: (1) presence or absence of a recent inpatient hospitalization, (2) presence or absence of a recent suicide attempt, and (3) presence or absence of recent self-harm.

#### 1.3.6. Intimate partner violence and sexual revictimization

At T2–T5, clinicians reported if their patients had experienced sexual revictimization, physical intimate partner violence (IPV), and emotional IPV within the previous six months, either as a victim only, perpetrator only, or victim–perpetrator. Each victimization variable was dichotomized to indicate its presence or absence. These variables grouped those who were victims, perpetrators, and victim–perpetrators.

### 1.4. Analyses

Some longitudinal variables were not measured at T1, and/or some T1 variables measured longer time periods (i.e. adult lifetime) than six-month time frames (as was measured at T5); thus, comparisons between T2 and T5 were used in longitudinal analyses. We used discriminant analyses (DFA) to evaluate whether composite patient stressor scores could classify study data point, which determines whether stressor scores significantly changed from the six months follow-up (T2) to the six years follow-up (T5). We used DFA to assess if QOL combined with GAF scores could classify study data point.

DFA combines multiple continuous variables into a linear composite predictor used to classify cases into dichotomous categories (i.e. T5 vs. T2) and offers follow-up univariate ANOVAs to see how individual predictors contributed to the model. Sample sizes for the DFA models include any participants who provide predictor data (i.e. those who provided only T2 data, only T5 data, or both), yielding varied sample sizes for the DFA models. We used a multivariable analysis of covariance (MANCOVA) to follow-up on the DFA models while controlling for patients’ treatment stage.

We used chi-squared tests to assess the association between study time point (T2 vs. T5) and patient victimization (sexual revictimization, physical IPV, emotional IPV). As chi-square requires two dichotomous variables (i.e. victim status and study time point), only T2 and T5 data were compared.

Lastly, we used paired samples *t*-tests and Wilcoxon-signed rank tests to assess differences in patient inpatient hospitalization and self-harm within the previous six months, as well as suicide attempts within the last year, at each time point (T1–T5); *t-*test and Wilcoxon-signed rank test analyses allowed for multiple pairwise comparisons between each time point. Given that patient hospitalization and suicide data did not meet assumptions of normality, both parametric and non-parametric approaches were used.

## 2. Results

### 2.1. Descriptive statistics

Table 2 includes descriptive data at T2 and T5. Thirty-nine (38.2%) of therapists’ patients were no longer in treatment. The most common reason for termination was objective, external factors (e.g. relocation, financial constraints, medical illness; 38.5%) followed by subjective, psychological factors (e.g. the patient ended treatment prematurely or felt treatment was not helping; 23.1%); successful resolution of treatment without full integration of self-states (12.8%); successful resolution of treatment with full integration (12.8%); death due to medical problems (5.1%); and death due to medical problems likely exacerbated by poor self-care (2.6%).

**Table 2.** Descriptive statistics for patient stressors, global assessment of functioning, and quality of life scores.

	Time 2					Time 5				
Variable	*N*	*M* (*SD)*	Median	Skew	Range	*N*	*M* (*SD)*	Median	Skew	Range
Victimization Stressors	26	2.31 (3.47)	1.50	2.67	0–15	50	1.02 (1.97)	.00	3.05	0–11
Family Stressors	26	6.04 (3.64)	6	.10	0–14	50	4.10 (3.01)	3	.61	0–11
Therapy Stressors	26	2.31 (2.48)	2	1.14	0–9	50	1.36 (1.72)	1	1.33	0–6
Resistance Stressors	26	5.97 (2.44)	6.50	−.52	0–10	50	4.08 (2.66)	4	.19	0–10
Resource Stressors	26	9.23 (3.27)	10	−.11	4–15	50	7.92 (4.35)	8	−.06	0–16
Total Stressors	26	23.54 (6.66)	23	−.24	10–37	50	17.16 (9.38)	17	.28	0–38
Global Assessment of Functioning*	51	8.06 (2.82)	8	.08	3–15	60	8.38 (3.18)	8	.26	3–15
Quality of Life	51	3.86 (1.69)	4	.15	2–7	60	6.13 (1.24)	6	.01	4–9

### 2.2. Patient safety over time

#### 2.2.1. Patient hospitalizations

One patient (1.6%) was admitted for an inpatient hospitalization that lasted for 28 days. Paired samples *t*-tests compared patient hospital admissions at each time point in the study (T1–T5). There were significantly fewer hospitalizations at T5 (*M* = .02, *SD *= .14) compared to T1 (*M* = .13, *SD *= .34), *t*(54) = 2.57, *p* < .05, Cohen’s *d* = .43. This significant finding replicated through bootstrapping. Non-parametric tests via Wilcoxon-signed rank test also demonstrated significant differences in patient hospitalization at T1 as compared to T5, *Z* = −2.45, *p* < .05.

#### 2.2.2. Patient suicide attempts

One therapist reported that his/her patient had made a suicide attempt over the previous six months (3.3%). Paired samples *t*-tests found no significant differences in number of suicide attempts over time. Non-parametric tests via Wilcoxon-signed rank test did not demonstrate any significant differences in number of suicide attempts between T1–T5.

#### 2.2.3. Patient self-harm incidents

Twenty-three therapists (43%) indicated that their patients had not engaged in self-harm over the previous six months. Twelve (20%) reported that self-harm occurred ‘very rarely,’ two (3.3%) reported it occurred ‘occasionally’ (i.e. approximately once per month), and three (5%) reported self-harm occurred ‘frequently’ (i.e. several times per month). No therapists reported that their patients were engaging in daily self-injurious behaviour. No significant differences in self-harm incidents over time were found.

### 2.3. Patient victimization

One therapist reported that his/her patient had experienced sexual revictimization (1.6%) and another therapist (1.6%) reported that his/her patient was a victim of physical IPV within the previous six months. Nine therapists (14.8%) indicated that they were unclear as to whether their patients were involved in emotionally abusive relationships. Eight reported that their patients were victims of emotional abuse (13.1%), and one therapist reported that his/her patient was both a victim and a perpetrator of emotional abuse (1.6%).

#### 2.3.1. Patient victimization over time

Chi-squared tests found a significant association between sexual revictimization and study time point, *X* ^2^(1) = 107.05, *p* < .001. The odds of experiencing recent sexual revictimization were 60 times higher at T2 then at T5 [95% CI: 8.59–419.02]. No associations were found between time points and physical or emotional IPV.

### 2.4. Patient quality of life and global assessment of functioning

Therapists reported considerable patient difficulties in romantic relationships. They described 28 patients (46%) as having ‘very poor, unstable, or absent’ romantic relationships. However, they rated 18 patients’ relationships (29.5%) at a 4 or 5, with 5 representing ‘loving and stable’ relationships. Friendships were more stable, with 28 (45.9%) rated as ‘loving and stable.’ Most experienced interference with occupational functioning. Only 11 patients were described as ‘working to full potential’ (18%) and 23 patients (37.7%) were unable to keep a job or were receiving disability. No therapists rated their patients’ GAF below a 3 (on a scale from 1–10), with 3 or lower representing an inability to function in almost all areas and possibility presenting harm to themselves and/or others. The majority of patients were reported as having a GAF of 6 (moderate symptoms and/or moderate functional impairment; *N* = 21; 35%).

#### 2.4.1. Quality of life and global functioning over time

The discriminant model using QOL composite scores and GAF scores to classify patients’ time point in the study (T2 vs. T5) was significant, *Χ^2^*(2) = 59.27, *p *< .001, canonical *r *= .65, *N* = 111. The model accounted for 65% of the variance in predicting patients’ study time point and correctly classified 81% of cases. Follow-up univariate ANOVAs indicated only GAF scores as a significant predictor in the model and that GAF scores were significantly higher at T5 vs. T2 (Table 3). Additionally, bootstrapping demonstrated that GAF scores were a robust predictor in the model.

**Table 3.** Univariate ANOVAs of patient stressor scores, global functioning, and quality of life and time point in study.

Discriminant Analysis*			MANCOVA (controlling for treatment stage)*		
Variable	*N*	*F* (df)	Canonical Coefficient	*F* (df)	Mean Square
Victimization Stressors	76	4.26 (1, 74)*	.81	4.23 (1, 73)*	28.48
Family Stressors	76	6.15 (1, 74)*^	1.09	6.13 (1, 73)*	63.63
Therapy Stressors	76	3.80 (1, 74)	.75	3.88 (1, 73)	15.06
Resistance Stressors	76	9.02 (1, 74)*^	1.09	9.40 (1, 73)*	59.66
Resource Stressors	76	1.82 (1, 74)	.20	1.95 (1, 73)	28.12
Total Stressors	76	9.51 (1, 74)*	−1.51	10.10 (1, 73)*	684.54
Global Assessment of Functioning	110	66.50 (1, 109)*	−.46	77.85 (1, 107)*	147.99
Quality of Life	110	.32 (1, 109)*	1.13	.56 (1, 107)	4.97

* *p* < .05

^Replicated through bootstrapping

#### 2.4.2. Controlling for patient treatment stage

The MANCOVA model assessing differences in QOL scores and GAF scores at different time points (T2 vs. T5) controlling for treatment stage was significant, *F*(2, 106) = 43.99, *p* < .001, ᶯ^2^ = .45, *N* = 110. Follow-up univariate ANOVAs indicated that GAF scores were significantly higher at T5 than T2, *F*(1) = 77.85, *p* < .001, ᶯ^2^ = .42 (Table 3).

### 2.5. Patient stressors

*Revictimization stress* for sexual assault and physically abusive relationships was absent among nearly all patients (98.1%), consistent with the low reporting of victimization. Two therapists (3.8%) reported that emotional abuse had a highly negative impact on patients. In *resource-related stressors*, finance- and health-related stressors caused a moderate level of stress (i.e. score of 3) for 25 and 40% of patients, respectively, while housing and work/school/volunteering caused less reported stress (66.67 and 48.3% ‘not at all’, respectively). Over half of therapists reported that stressors from marriages and children (i.e. *family-related stressors*) were absent (52.5 and 54.2%, respectively). Families of origin caused moderate stress for 32.2% of patients.

#### 2.5.1. Patient stressors over time

The discriminant model using the six composite patient stressor scores to classify patients’ time point in the study (T2 vs. T5) was significant, *Χ^2^*(6) = 18.76, *p *< .01, canonical *r *= .48, *N* = 76. The model accounted for 48% of the variance in predicting time point, correctly classifying 78% of cases. Univariate ANOVAs indicated that revictimization, family, and resistance-related stressor scores, as well as the total stressor score, were significant predictors in the model, and each of these stressors was significantly lower at T5 (Table 3). Bootstrapping indicated that family and resistance-related stressors were robust predictors.

#### 2.5.2. Controlling for treatment stage

The MANCOVA model assessing differences in patient stressors at T2 vs. T5, controlling for treatment stage, was significant, *F*(6,68) = 3.55, *p* < .01, ᶯ^2^ = .24, *N* = 76. Follow-up univariate ANOVAs indicated that stressor scores related to victimization, family, resistance, and the total stressors were significantly lower at T5 (Table 3).

## 3. Discussion

The present study examines patients’ functioning six years after enrolment in the TOP DD study. At T5 as compared to T2, GAF was significantly higher and stressors, particularly related to family relationships and conflict among self-states, were significantly lower, even when controlling for treatment stage. There were also significantly fewer hospitalizations at the follow-up time point than at the beginning of the study. These findings indicate that patients continued to demonstrate improved functioning and had less need for intensive and costly intervention in the form of hospitalization. No outcome assessed in this study worsened over time.

The decreased need for hospitalization is especially notable; there is an immense economic cost associated with trauma and for patients with DD in particular (Brand, Lanius, Vermetten, Loewenstein, & Spiegel, 2012; Ferry et al., 2015; Myrick, Webermann, Putnam, & Brand, in press). Our results support the view that the phasic trauma treatment model for DD leads to reduction in the burden of disease for patients. Further, these results are consistent with prior studies that this treatment model is associated with substantial cost savings, largely due to reduced need for treatment at more intensive, costly levels of care (Brand, Loewenstein, & Spiegel, 2014). Preliminary analyses using TOP DD data show that both hospitalization and outpatient psychotherapy costs decreased significantly over time (Webermann, Brown, Brand, Loewenstein, & Putnam, 2013).

Suicide attempts and parasuicidal behaviour were not significantly different six years into treatment among this subset of the TOP DD sample, although both showed significant reduction in the larger TOP DD sample (Brand et al., 2013). In this subsample, suicide attempts were low from the beginning of the study (T1), which suggests that almost all of these patients were successfully maintaining safety, yet such low rates of suicide mean that this outcome is unlikely to improve due to a floor effect. Similarly, less than 10% of patients in the sample were engaging in self-harm once a month or more, and none were engaging in daily self-harm; differences would be difficult to detect. In contrast, in the overall TOP DD sample, at T1, self-harm was more common (19% engaged in self-harm on average once per month or more and 16% made a suicide attempt within the previous year), which is likely why earlier analyses with the larger TOP DD sample found improvements in both outcomes. It is possible that this subsample has better impulse control and/or is able to develop and maintain a better alliance than the overall sample, although those who participated in T5 did not have different levels of mistrust of their therapists or treatment teams at T2 than those the current subsample.

Despite patients’ GAF improving over time, many continued to be negatively affected by daily stressors and showed serious difficulty managing healthy relationships, in line with Lampe et al.’s (2014) findings. Nevertheless, IPV was low and sexual revictimization declined. Similarly, family-related stressors decreased over six years in treatment, as did internal conflict between self-states. Interpersonal difficulties are substantial stressors for DD patients (Brand et al., 2009; Brand, Lanius, et al., 2012), perhaps because childhood sexual abuse and dissociation are associated with poor social cognition (Nazarov et al., 2014). Marked internal conflict among self states is an indicator of poorer prognosis in this population (Kluft, 1994). Significant reduction in these difficulties indicates that these patients are progressing in treatment, gaining stability, and making interpersonal and intrapersonal improvements.

Clinicians must be mindful of attachment difficulties common among DD patients and be attentive to ways in which these difficulties manifest. DD experts strongly recommend focusing on attachment as it pertains to the therapeutic alliance as well as the development of healthy relationships, throughout all stages of treatment (Brand et al., 2012). Few patients were highly stressed by mistrust or conflict with the treatment team, which is notable, given the childhood maltreatment and attachment difficulties endemic among this group (Foote et al., 2006; Johnson et al., 2006; Sar et al., 2007). This may suggest that clinicians and patients were generally successful in noticing and managing patients’ traumatic transference and attachment difficulties (Loewenstein, 1993; Kluft, 1990).

### 3.1. Limitations

There are notable limitations to the study, primarily centring on the small sample, which may be a particularly motivated sample of patients or a skilled sample of clinicians. There was a high attrition throughout the TOP DD study, and the current study did not obtain patient-reported data. Due to small sample size, some variables were dichotomized, preventing more detailed analyses of nuanced treatment concerns, such as how self-harm is linked to IPV. Finally, we did not examine patients’ time in treatment prior to the TOP DD study or alliance, thus some of our inferences are limited.

### 3.2. Future research and conclusion

Future longitudinal research should obtain self-report data from DD patients, as well as clinicians, and seek to find methods for improving retention in DD treatment outcome studies. This study demonstrates that DD patients continue to show a range of improvements during six years of dissociation-focused, phasic treatment. Therapists’ reports indicated significant improvements in patients’ global functioning; reductions in stress related to family relationships and internal conflict among self-states; and decreased sexual revictimization. Additionally, patients required significantly fewer hospitalizations. Despite improvements, patients continued to experience difficulties in their romantic relationships, friendships, and occupational functioning. Findings provide support for the staged model of complex trauma treatment. However, it is clear that treatment of severe DD patients must address their profoundly damaged capacity for relationships (Freyd, 1996; Herman, 1992; Putnam, Harris, Lieberman, Putnam, & Amaya-Jackson, 2015). Research needs to continue studying trauma-focused DD treatments and their economic and psychosocial impact.

## Highlights

- Until recently, there has been a paucity of longitudinal treatment research on dissociative disorder (DD) patients.
- Sixty-one therapists who participated in the initial phase of the Treatment of Patients with Dissociative Disorders (TOP DD) study answered questionnaires about their study patient’s stressors, quality of life, global functioning, victimization, and safety.
- Longitudinal analyses demonstrated patients had significantly fewer stressors, instances of sexual revictimization, and psychiatric hospitalizations.
- Longitudinal analyses demonstrated that patients were functioning at a higher level overall.
- Despite marked initial difficulties and functional impairment, DD patients benefit from specialized treatment.

## Supplementary Material

Supplementary material

## Note

1. We use the term ‘self-state’ to refer to dissociated states which are also referred to as identities, parts, personalities, and alters.
