# Supplementary material for: Six-year follow-up of the treatment of patients with dissociative disorders study*
Source: Eur J Psychotraumatol. 2017 Jun 28;8(1):1344080. doi: 10.1080/20008198.2017.1344080 (PMC5492082; doi:10.1080/20008198.2017.1344080)
Supplement: Supplementary material [file zept_a_1344080_sm7167.docx]

| **Citation** | **Objective** | **Summary of Major Findings** |
| --- | --- | --- |
| Brand, B. L., Classen, C. C., Lanius, R., Loewenstein, R. J., McNary, S. W., Pain, C., &  Putnam, F. W. (2009). A naturalistic study of dissociative identity disorder and dissociative disorder not otherwise specified patients treated by community clinicians. *Psychological Trauma: Theory, Research, Practice, & Policy, 1*(2), 153-171. | To examine if outpatient treatment provided by community clinicians is associated with reductions in psychiatric symptoms. | - DD patients reported very severe symptoms and their therapists reported low functioning (GAF scores), as compared to patients with other psychiatric disorders. - DD patients in later stages of treatment had less severe symptoms and better daily functioning than those in the earlier stages of treatment. - DD patients in the last stage of treatment still suffered from higher levels of symptoms than patients with other psychiatric disorders. |
| Brand, B. L. & Loewenstein, R. J. (2014). Does phasic trauma treatment make patients with dissociative identity disorder Treatment more dissociative? *Journal of Trauma & Dissociation. 15*, 52-65. | To examine the speculation put forward by some authors that phasic trauma treatment makes DD patients worse or is ineffective in reducing symptoms. | - Patients reported a significant decrease in identity alteration and hearing the voices of their dissociated self-states over time in DD treatment. - Amnesia scores showed a trend toward decline over time in DD treatment. - GAF scores, as reported by therapists, increased over the course of treatment, suggesting that patients became more functional over time. - Results of this study challenged the notion that DD treatment makes patients worse. |
| Brand, B. L., McNary, S. W., Myrick, A. C., Loewenstein, R. J., Classen, C. C., Lanius, R. A., Pain, C., & Putnam, F.W. (2013). A longitudinal, naturalistic study of dissociative disorder patients treated by community clinicians. *Psychological Trauma: Theory, Research, Practice, & Policy, 5*(4), 301-308. | To examine outcomes in community based DD treatment over 30-months. | - Patients’ reports of dissociative symptoms and general psychiatric symptoms significantly decreased over the 30-month duration of the study. - Patients’ reports of chronic pain, self-harm, dangerous and impulsive behavior, and using prescription or illicit drugs significantly decreased over time in treatment. - Therapists reported that self-harm, suicide attempts, and hospitalizations decreased over time in treatment. |
| Brand, B. L., Myrick, A. C., Loewenstein, R. J., Classen, C. C., Lanius, R., McNary, S. W., Pain, C., & Putnam, F. W. (2011). A survey of practices and recommended treatment interventions among expert therapists treating patients with dissociative identity disorder and dissociative disorder not otherwise specified. *Psychological Trauma: Theory, Research, Practice, & Policy, 4,* 490-500. | To examine different treatment techniques that are recommended by DD experts at various stages of DD treatment. | - In early treatment, experts recommended the following interventions most frequently: diagnosing disorders; establishing safety; stabilizing from current day stressors/crises; improving the therapeutic alliance; teaching/practicing grounding, containment, and self-care; providing psychoeducation; and developing healthy relationships, affect tolerance, and impulse control. - During the middle stage of treatment, experts recommended the following interventions most frequently: stabilizing from current stressors and crises; teaching/practicing containment, grounding and awareness of body sensations; developing affect tolerance, impulse control and cooperation with parts; identifying and working with parts; improving the therapeutic alliance; and using CBT focused on modifying distorted cognitions. - There was less uniformity among experts in the last stage of treatment. Those interventions recommended with the highest frequency included developing healthy relationships; awareness of emotion and body sensation and self-care; stabilizing from daily stressors and crises; processing reactions to therapy and the improving the alliance; using ego-strengthening activities; and stabilizing following intrusions from alleged perpetrators. |
| Brand, B. L., & Stadnik, R. (2013). What contributes to predicting change in treatment of dissociation: Initial levels of dissociation, PTSD, or overall distress? *Journal of Trauma & Dissociation, 14*, 328-341. | To examine whether the changes in symptomology, including PTSD and general distress, were related to the decrease of dissociative symptoms during treatment. | - Initial total dissociation was associated with PTSD and general distress. Change in total dissociation over the 30-month time-span of the study was associated with greater change in PTSD symptoms and general distress. - Improvement in dissociative symptoms was correlated with greater change in other symptomology. - Initial total dissociation was the best predictor of change in DD symptoms. |
| Cronin, E., Brand, B. L., & Mattanah, J.F. (2014). The impact of the therapeutic alliance on treatment outcome in patients with dissociative disorders. *European Journal of Psychotraumatology.* http://dx.doi.org/10.3402/ejpt.v5.22676 | To examine the relationship between therapeutic alliance and treatment outcome among patients with DD. | - Patient and therapist alliance was moderately correlated with treatment outcome, including lower symptoms and better overall functioning. - Patients’ reports of the alliance were more strongly predictive of treatment outcomes than were therapists’ reports of the alliance. |
| Engelberg, J. C., & Brand, B. L. (2012). The effect of depression on self-harm and treatment  outcome in patients with severe dissociative disorders. *Psi Chi Journal of Psychological Research, 17*, 115-124. | To examine if DD patients that had severe depression were more likely to self-harm or attempt suicide than DD patients that had less severe depression. | - Severe depression was associated with more self-harm and suicide attempts. - Patients with DDs who suffered severe depression tended to exhibit more self-harm and suicide attempts, which suggests that targeting depressive symptoms directly could be helpful in reducing self-harm or number of suicide attempts. - The study found partial support for the hypothesis that patients with severe depression would show less improvement compared to less severely depressed patients. |
| Myrick, A. C., Brand, B. L., McNary, S. W., Classen, C. C., Lanius, R. A., Loewenstein, R. J….Putnam, F. W. (2012). An exploration of young adults' progress in treatment for dissociative disorder. *Journal of Trauma & Dissociation, 13*, 582-595. | To compare a group of young adults with DDs (ages 18-30) to a group of older adults with DDs (ages 31+). | - The young adult subsample entered treatment with higher dissociative symptoms and lower adaptive capacities than older adults. - Rates of self-injury and suicidality seemed to decrease at a faster rate for the young adults as compared to the older adults. None of those in the young adult sample reported a suicide attempt in the fourth and final study follow-up. - Dissociative symptoms decreased, but remained clinically significant throughout the study. PTSD and general distress symptoms also remained high and decreased at a slower rate. Hospitalizations remained high for those in the young adult subsample. - This study’s results suggest that younger DD patients respond more quickly to treatment than older DD patients. |
| Myrick, A.C., Brand, B.L., & Putnam, F.W. (2013). For better or worse: The role of  revictimization and stress in the course of treatment for dissociative disorders. *Journal of Trauma & Dissociation, 14*(4), 375-389.doi: 15299732.2012.736931. | To examine the relationship between life stressors, revictimization, and treatment outcome. | - Across the DD sample, rates of revictimization decreased over time. Emotional abuse was the most frequent stressor related to victimization in either category. - Those with ‘worsening’ symptoms reported the highest amount of revictimization, with sexual revictimization being the most common type. - Patients in the ‘improving’ group had fewer stressors than those in the worsening group. - Patient resistance and distrust of the treatment team were associated with worsening symptoms. |
| Myrick, A. C., Chasson, G. S., Lanius, R. A., Leventhal, B., & Brand, B. L. (2015). Treatment of  complex dissociative disorders: A comparison of interventions reported by community therapists versus those recommended by experts. *Journal of Trauma & Dissociation, 16*(1), 51-67. | To compare interventions used by a group of community therapists to those recommended by experts of trauma treatment. | - Community therapists and DD expert clinicians reported using similar treatment techniques, including the necessity of working with different self-states and improving cooperation among self-states. - Community therapists reported significantly less focus on relationally-oriented interventions, teaching and using grounding and containment skills, and stabilizing patients after revictimization. Patients may benefit from therapists increasing their use of these interventions. |
| Webermann, A. R., Brand, B. L., & Chasson, G. S. (2014). Childhood maltreatment and intimate partner violence in dissociative disorder patients. *European Journal of Psychotraumatology, 5*. doi: 10.3402/ejpt.v5.24568 | To examine the association between childhood maltreatment and IPV among DD patients, who evidence risk factors for IPV. | - According to therapists, DD patients evidenced high rates of adult intimate partner victimization, with 1/3 experiencing physical IPV and over ½ experiencing emotional IPV. - The odds of physical IPV were 14 times greater among DD patients with histories of physical and emotional childhood maltreatment. - The odds of emotional IPV were 3 times greater among DD patients with histories of childhood neglect and exposure to familial domestic violence. - The study was the first to provide evidence of a developmental trajectory between childhood maltreatment and IPV among DD patients, as has been found in other clinical and non-clinical populations. - The results suggest DD clinicians should regularly screen DD patients for past or current IPV. |
| Webermann, A. R., Myrick, A. C., Taylor, C. L., Chasson, G. S., & Brand, B. L. (2015). Dissociative,  depressive, and PTSD severity as correlates of non-suicidal self-injury and suicidality in  dissociative disorder patients. *Journal of Trauma & Dissociation, 17*(1), 67-80. | To examine symptom severity as a predictor of DD patient self-harm and suicidality, focusing specifically on dissociative, depressive, and PTSD symptoms. | - As compared to the extant literature on self-harm and suicidality among DD patients, those in the TOP DD study (all of whom were in consistent treatment) evidenced lower rates of suicide attempts, while they self-harmed at comparable rates to DD patients in other studies. - Dissociative symptom severity predicted patients who evidenced recent self-harm and suicide attempts. - Depression symptom severity predicted recent self-harm, but not recent suicide attempts. - The results provide credence to expert guidelines on the treatment of DDs which suggest targeting dissociative symptoms at the start of treatment, as a means of promoting patient safety and stability. |

^1^ Abbreviations (in order of appearance in table): DD = dissociative disorders; GAF = global assessment of functioning; DID = dissociative identity disorder; CBT = cognitive behavioral therapy; PTSD = posttraumatic stress disorder; IPV = intimate partner violence
